# Single-molecule imaging reveals how mavacamten and PKA modulate ATP turnover in skeletal muscle myofibrils

**DOI:** 10.1085/jgp.202213087

**Published:** 2022-11-17

**Authors:** Matvey Pilagov, Laurens W.H.J. Heling, Jonathan Walklate, Michael A. Geeves, Neil M. Kad

**Affiliations:** 1 School of Biological Sciences, University of Kent, Canterbury, UK

## Abstract

Muscle contraction is controlled at two levels: the thin and the thick filaments. The latter level of control involves three states of myosin heads: active, disordered relaxed (DRX), and super-relaxed (SRX), the distribution of which controls the number of myosins available to interact with actin. How these are controlled is still uncertain. Using fluorescently labeled ATP, we were able to spatially assign the activity of individual myosins within the sarcomere. We observed that SRX comprises 53% of all heads in the C-zone compared with 35% and 44% in the P- and D-zones, respectively. The recently FDA-approved hypertrophic cardiomyopathy drug, mavacamten (mava), significantly decreased DRX, favoring SRX in both the C- and D-zones at 60% and 63%, respectively. Since thick filament regulation is in part regulated by the myosin-binding protein-C (MyBP-C), we also studied PKA phosphorylation. This had the opposite effect as mava, specifically in the C-zone where it decreased SRX to 34%, favoring DRX. These results directly show that excess concentrations of mava do increase SRX, but the effect is limited across the sarcomere, suggesting mava is less effective on skeletal muscle. In addition, we show that PKA directly affects the contractile machinery of skeletal muscle leading to the liberation of repressed heads. Since the effect is focused on the C-zone, this suggests it is likely through MyBP-C phosphorylation, although our data suggest that a further reserve of myosins remain that are not accessible to PKA treatment.

## Teaser

Control of muscle contraction is still not understood. Here, we image fluorescently tagged ATP with single-molecule precision to reveal the activity of individual myosins in different sarcomeric locations. We show how treatment with PKA and the FDA-approved drug mavacamten affects relaxed myofibrils.

## Introduction

**Figure S1.**
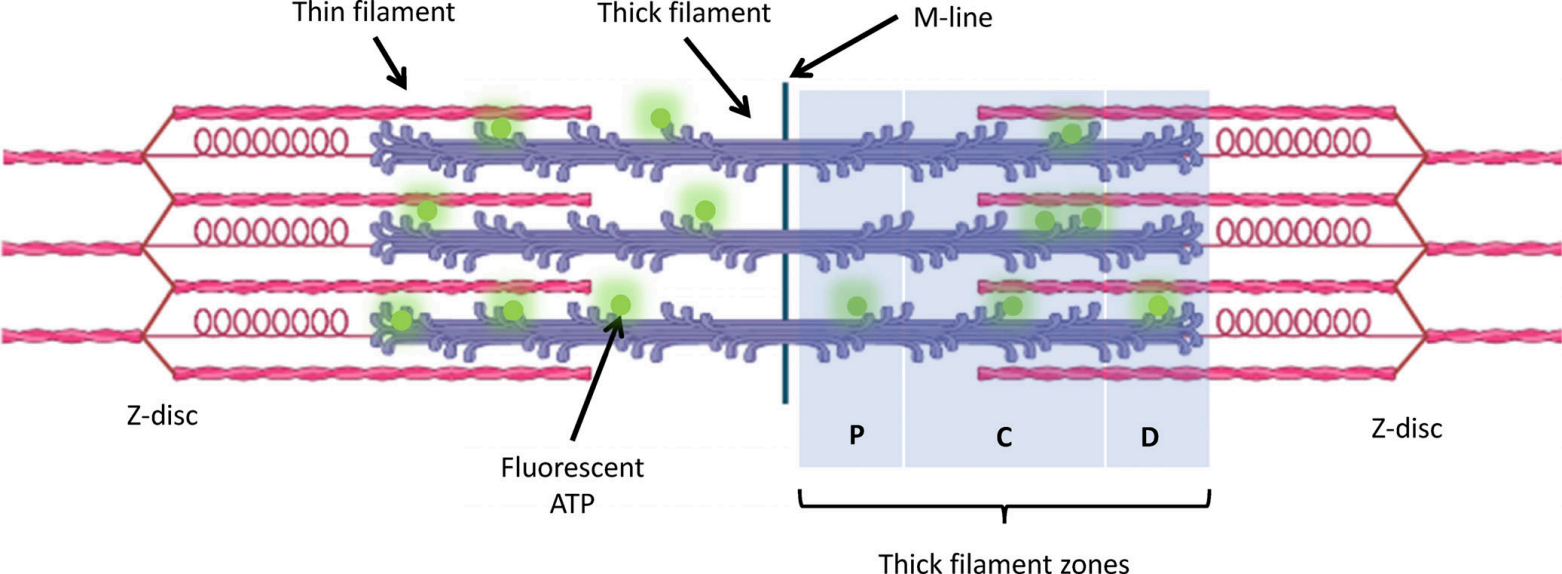
**The structure of a sarcomere.** A schematic representation of a sarcomere with Cy3-ATP (green) attachments to the myosin-containing thick filaments (purple). The thin filament is shown in pink and is decorated with troponin and tropomyosin (not shown). The thick filament can be subdivided into P-, C-, and D-zones, depending on their MyBP-C occupancy. The M-line is centrally located between the terminal Z-discs. Please also see Fig. 2 a. Created using BioRender.com.

Striated muscle contraction requires the coordinated action of molecular motors. This is mediated by high levels of organization starting with the minimal contractile unit: the sarcomere (Fig. S1). Each sarcomere is organized into thick filaments, comprised mostly of myosin, and actin-containing thin filaments. At the center of the sarcomere lies the myomesin containing the M-line, and radiating laterally from this are the two halves of the bipolar thick filament. The ends of each sarcomere are delineated by the Z-discs from which thin filaments extend toward the M-line, overlapping with myosins on the thick filament. This overlap permits the thick and thin filaments to slide over one another, driven by the ATP-powered motor protein myosin II, resulting in contraction. The thick filament can be further subdivided into three zones, P, C, and D, outward from the M-line. The defining feature of the C-zone is myosin binding protein-C (MyBP-C), which occurs in seven to nine stripes along each half of the thick filament (Bennett et al., 2020). To ensure coordinated action of multiple sarcomeres, these are organized into long linear arrays stacked together into a 3-D lattice to form myofibrils—the contractile organelle of myocytes.

Activation of muscle contraction requires calcium release into the myocyte, which results in the motion of tropomyosin on the thin filament to expose sites for myosin to bind. This rapid response to stimulus controls contraction on a beat-to-beat basis in cardiac muscle, as well as enables skeletal muscle contraction. However, to fine-tune the force, a second mechanism of activation exists to control the number of heads available on the thick filament for contraction (Irving, 2017). Force is affected because it is the product of the force per myosin head and the number of heads available (Spudich, 2019). Thick filament regulation improves contractile efficiency, preventing heads from hydrolyzing ATP unnecessarily. Many factors control thick filament regulation, including direct phosphorylation of the regulatory light chain, the amount of resistive force experienced, and phosphorylation of MyBP-C (Heling et al., 2020). These and other factors alter the equilibrium between three myosin states of declining ATPase activity: active, disordered relaxed (DRX; McNamara et al., 2016), and super-relaxed (SRX; Stewart et al., 2010; Hooijman et al., 2011). The precise nature of these three states remains an open question; however, it is clear that in fluorescent-ATP chase experiments using relaxed myosins and myofibrils (Hooijman et al., 2011; Walklate et al., 2022), two kinetic phases are present, the faster of which is DRX, and its ~10-fold slower counterpart is SRX. In addition, the structural correlates of these states are also not certain, with evidence both for and against the SRX state being folded onto the thick filament backbone (Caremani et al., 2019; Chu et al., 2021; Schmid and Toepfer, 2021; Craig and Padron, 2022) Cardiac MyBP-C (cMyBP-C) has been shown to directly bind myosin and push the equilibrium to favor SRX and reduce force. However, cMyBP-C can bind the thin filament and activate contraction (Inchingolo et al., 2019); therefore, cMyBP-C can act as both activator and repressor in the sarcomere (Heling et al., 2020). Phosphorylation of cMyBP-C with the β-adrenergic-activated cAMP-dependent protein kinase (PKA) leads to a reduction in its repressive activity, tuned by the amount of phosphorylation. Therefore, phosphorylation might reconcile these two activities.

Hugh Huxley and Jean Hanson stated in their landmark 1954 paper (Huxley and Hanson, 1954) “Myofibrils…are admirable objects for high-resolution microscopy”, and our studies aim to build on their vision.

Here, we studied the activity of myosins in the organized structure of rabbit psoas myofibrils at the single-molecule level. By imaging fluorescent-ATP binding and releasing the within myofibrils, we were able to directly measure individual myosins utilizing ATP within a sarcomere with a high spatial precision (Nelson et al., 2020). We found that the amount of repressed myosin, i.e., SRX is relatively even across the thick filament. Upon challenge with the pharmacological agent mavacamten (mava), we observed a switch from DRX to SRX in both the C- and D-zones, particularly pronounced in the D-zone. Upon PKA-phosphorylation, only the C-zone showed a significant switch back to DRX from SRX, increasing the number of heads available for active contraction. This implies that PKA mediates its action through MyBP-C phosphorylation, which is only present in the C-zone, although it is possible that other proteins may be the target. These activation and repression effects are not complete, indicating that a reserve of myosin exists beyond the reach of PKA activation and mava suppression. For the latter, this confirms the specificity of action for this drug; however, for the former, another factor is likely involved with activating skeletal muscle myosin even in the presence of MyBP-C phosphorylation.

## Materials and methods

### Preparation of myofibrils

Flash-frozen rabbit psoas muscles strips with a diameter of 1–2 mm were fully immersed in four 30-min changes of ice-cold fiber preparation buffer (6 mM imidazole, 8 mM Mg-acetate, 70 mM propionate, 5 mM EGTA, 7 mM ATP, and 1 mM Na-azide, pH 7.0). Skinning was then performed by soaking equilibrated samples for 2 h in a fiber preparation buffer with added 0.5% Brij 58 (P5884; Sigma-Aldrich). Skinned samples were stored in the fiber preparation buffer mixed 1:1 with ice-cold glycerol for 2 h before moving to −20°C for long-term storage.

Prior to myofibril preparation, the muscle strips were immersed in a wash buffer (6 mM Mg-acetate, 10 mM EGTA, 54 mM Na-acetate, 18 mM Na_2_SO_4_, 10 mM MOPS, 5 mM ATP, and 1 mM DTT, pH 7.4). The strips were separated into threads and transferred into a 2-ml tube containing 800 μl of wash buffer. Threads were twice homogenized using a Tissue Ruptor II with a rest period of 30 s (on ice). The myofibril concentration was kept constant by ensuring an OD_600_ of ∼0.8.

No live animals were used in these studies. Muscle tissue was collected in accordance with the U.K. Animals (Scientific Procedures) Act 1986 and associated guidelines.

### Imaging chamber construction

Imaging chambers were constructed using a microscope slide with two holes drilled through and onto this, a coverslip was placed and secured with a 180-µm thick gasket cut from double-sided tape. Both drilled slides and coverslips were cleaned prior to the assembly by shaking overnight in 100% ethanol. These were further cleaned using a plasma cleaner (Harrick Plasma PDC 32G 2) for 5 min. Plasma-cleaned coverslips were coated with 15 µg/ml poly-L-lysine and allowed to dry for 30 min.

### Imaging

Prior to imaging, the chamber was washed three times with 100 μl wash buffer and incubated for 30 min. Myofibrils were introduced through one drilled hole and allowed to attach to the surface for 30 min. To prevent nonspecific binding of subsequent reagents to the coverslip surface, 100 μl of 10 mg/ml BSA (A7906; Sigma-Aldrich) was washed into the chamber for 2 min followed by 100 μl wash buffer. Z-discs were labeled in situ with preconjugated (1 h) 200 nM Alexa-488 goat anti-mouse IgG (A11001; Thermo Fisher Scientific) and 200 nM anti-α-actinin mouse antibody (A7811; Sigma-Aldrich) for 1 h prior to washing with 100 μl wash buffer. Finally, 10 μl imaging buffer (1 mg/ml BSA, 5 nM phosphoenolpyruvate [PEP], 1 μl pyruvate kinase [PK], 6 mM Mg-acetate, 10 mM EGTA, 54 mM Na-acetate, 18 mM Na_2_SO_4_, 10 mM MOPS, 1 mM DTT, 5 nM Cy3-ATP, and 5 mM ATP, pH 7.4) was washed into the chamber and equilibrated for 10 min prior to imaging. Cy3-ATP was synthesized (Toseland and Webb, 2011) and provided by Dr. C.P. Toseland (University of Sheffield, Sheffield, UK).

When used, DMSO-solubilized mava (myk-461) was added into the imaging buffer at a final concentration of 30 µM and final DMSO < 1%. cAMP-dependent protein kinase (PKA; P2645; Sigma-Aldrich) was incubated with myofibrils for 1 h before washing into the imaging chamber.

### Stopped-flow experiments

A HiTech Scientific (SF-61 DX2) stopped-flow was used with 700 μl sample syringes. We used an LED lamp (290 nm; Ocean Optics) as the light source, and the tryptophan fluorescence output was measured using a WG320 filter. Samples reaching the observation cell were mixed in a 1:1 ratio. Stopped-flow analysis was used to determine ATP/ADP ratio for Cy3-ATP by mixing myosin head subfragment 1 (S1) with Cy3-ATP at a range of S1 concentrations (2.5 µM–39 nM) while keeping the Cy3-ATP concentration constant at 1 µM. Concentrations are premix values in stopped-flow buffer (25 mM KCl, 20 mM MOPS, 5 mM MgCl_2_, and 1 mM Na-azide, pH 7).

### ATP regeneration

**Figure 1.**
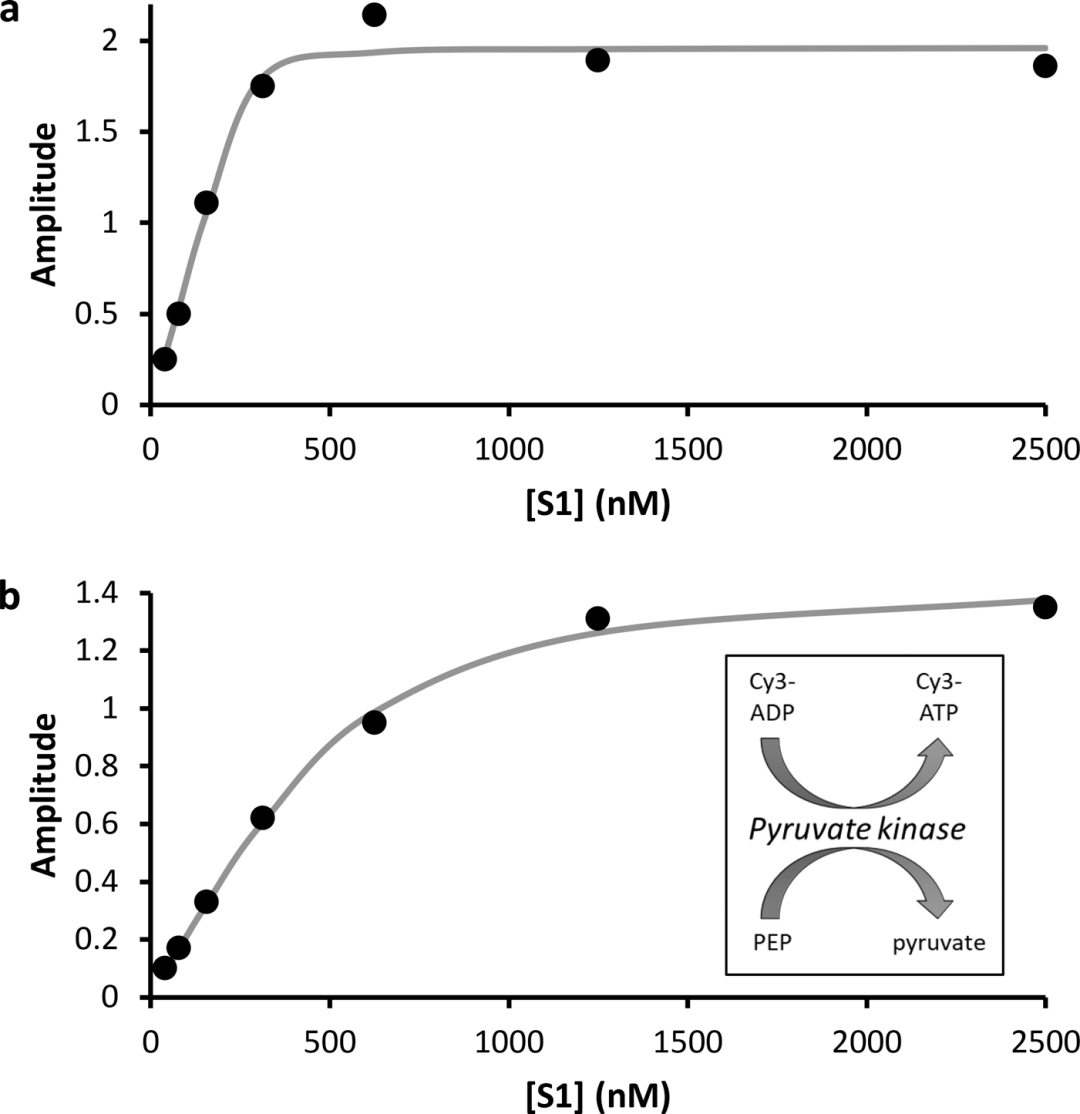
**Cy3-ATP binding to myosin. (a)** The amplitudes from stopped-flow transients measuring binding of Cy3-ATP to myosin indicate a 2:1 ratio of Cy3-ATP:myosin S1 in the absence of nucleotide regeneration. Fitted values: E_T_ 500 nM, S_T_ 278 (±38) nM, V_max_ = 1.96 (±0.08). **(b)** Using pyruvate kinase–based regeneration (inset), the binding of Cy3-ATP is seen to follow a binding isotherm consistent with a single ATP per myosin S1. Fitted values: E_T_ 500 nM, S_T_ 544 (±85) nM, V_max_ = 1.46 (±0.06). Each point was an average of at least three repeats.

ATP was regenerated using ∼1 unit of rabbit muscle PK (P0294; Sigma-Aldrich) and PEP (concentration equimolar to Cy3-ATP; Fig. 1). PK, PEP, and Cy3-ATP were incubated for at least 60 min on ice.

### Image acquisition

All single molecule experiments were conducted using a custom-built oblique angle fluorescent (OAF) microscope, described previously (Desai et al., 2015). Cy3-ATP was excited using a 561-nm laser (OBIS LS laser) at 20 mW, and Alexa-488-labeled antibodies were excited using an Oxxius 488 nm also at 20 mW. Images of Alexa-488-labeled Z-discs were acquired for the first 30 s after which the 488-nm laser was switched off and stroboscopic imaging with the 561-nm laser was initiated. During stroboscopic imaging, the laser on time was kept at 200 ms, while off times were changed depending on the desired overall time frame. This was achieved using a custom-designed Arduino-based control system, controlling both the laser and the camera exposures. At least three myofibrils were imaged per chamber.

### Data analysis

As described above, each video was comprised of two parts: the first 30 s corresponded to images of the Z-discs using fluorescent antibodies and the remainder was Cy3-ATP. Automated tracking of the positions for each fluorescent spot was achieved using the ImageJ plugin, TrackMate. The tracking parameters used for TrackMate included the estimated object diameter of 390 nm for the LoG detector, 100 nm for the linking and gap-closing distances used by the simple LAP tracker, and the gap-closing max frame gap was set to 20 frames and manually inspected to ensure the events were not split during automated tracking.

All tracks were then analyzed using Microsoft Excel. Based on the TrackMate-derived superlocalized positions of the bound Z-disc antibodies, it was possible to fit a straight line along each Z-disc. This was used to align the sarcomere being examined to the horizontal plane (see supplementary text at the end of the PDF). The Z-disc boundaries were accurately defined by binning the positions of the fluorescent antibodies along the now horizontal axis of the sarcomere and then fitting each Z-disc to a Gaussian distribution. This enabled a user-free determination of the center of each Z-disc; the accuracy was determined as 31.8 nm from the average SD of 1,318 Gaussian Z-disc fits. Importantly, this value provides the error in assigning the subsarcomeric zone boundaries. These boundaries (P-zone [0–159 nm], C-zone [160–500 nm], and D-zone [501–800 nm]) were measured from the M-line, which were defined midway between the Z-discs for each sarcomere. These values are subject to the same error as the determination of the Z-disc positions.

All bound Cy3-ATP localizations were then classified according to the zone in which they were found. To calculate the spatial precision for Cy3-ATP positioning, we adopted an unbiased empirical approach developed by Gelles et al. (1988). This approach compares the positions of static objects in the field of view over time and includes all sources of error without requiring any assumptions to be made about the hardware or software used. Assuming the objects are stationary, the SD of the relative positions defines the accuracy of the localization. We compared the positions of 75 Cy3-ATP localizations of between 10 and 21 frames in duration. The average SD, therefore, the spatial precision, was found to be 29.2 nm in X and 26.7 nm in Y using this method.

In addition to position, we recorded lifetimes binned at the frame time interval of the experiment being performed. For all data, except the determination of the photobleaching rate constant, this interval was 2 s. All data were fitted to the sum of three exponentials in Excel using the Solver GRG nonlinear engine with linear weighting. The fitted rate constant parameters were limited to a 1.8–2.2-fold difference from the global (uncorrected) values for the whole sarcomere. This ensured no overlap between rate categories (e.g., the SRX rate constant window did not overlap with the DRX window). To ensure the triple exponential fit was optimal, we examined the quality of the fit residuals looking for characteristics of underfitting, where the residuals would show a systematic inconsistency through time, and this was visually compared with double exponential fits. For overfitting, we would expect to observe duplication of fitted rate constants, but these were not observed. Standard errors for regression coefficients were obtained using Solver Statistics VBA (Billo, 2011). All subsequent arithmetic functions were performed with error propagation using standard a propagation formulae (Caldwell and Vahidsafa, 2022).

### Online supplemental material

**Figure S2.**
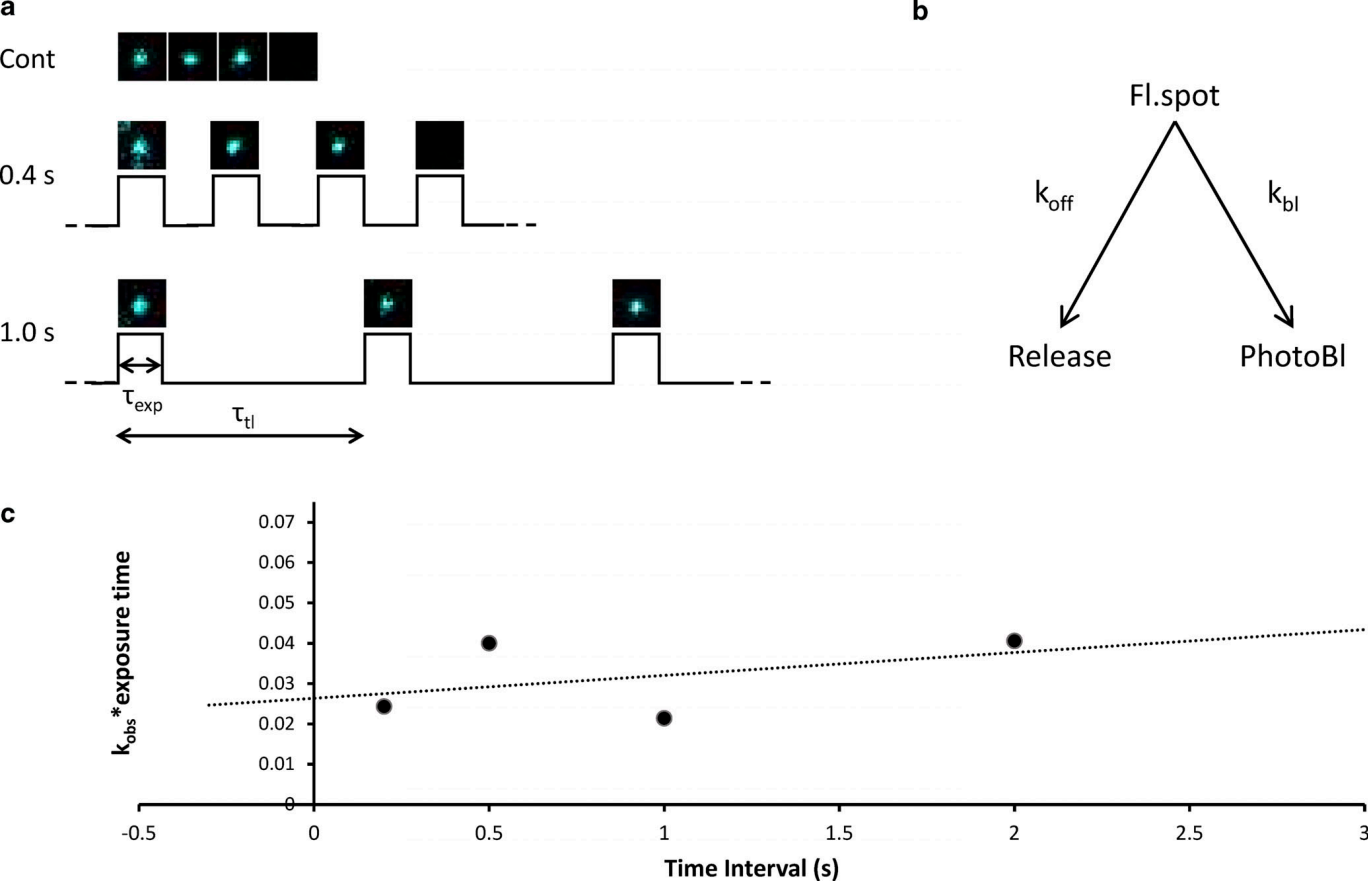
**Time lapse imaging reveals the contribution of photobleaching. (a)** Stroboscopic illumination enables the excitation energy to be spread out over time. In this example, a fluorophore disappears after three exposures; when under continuous illumination (top) this takes 0.6 s; however, if there is a 0.4 s gap between frames (middle), then the same exposure takes 1.2 s; when the time between exposures is further extended to 1 s (bottom), then only after 3 s will the fluorophore disappear. The images used are representative and not actual images recorded during such a stroboscopic image series. **(b)** The kinetics of this process are represented by two unidirectional rate constants, therefore the rate of loss of the fluorescent spot is *k*_*off*_ *+ k*_*bl*_. **(c)** Plot of the dependence of *k*_*obs*_ * *τ*_*exp*_ on the time lapse (*τ*_*tl*_) reveals a linear relationship from which the intercept provides the photobleaching rate constant (0.13 ± 0.05 s^−1^). Time lapses used were 200 ms (*n* = 1,689), 500 ms (*n* = 3,520), 1 s (*n* = 1,312), and 2 s (*n* = 1,455).

**Figure S3.**
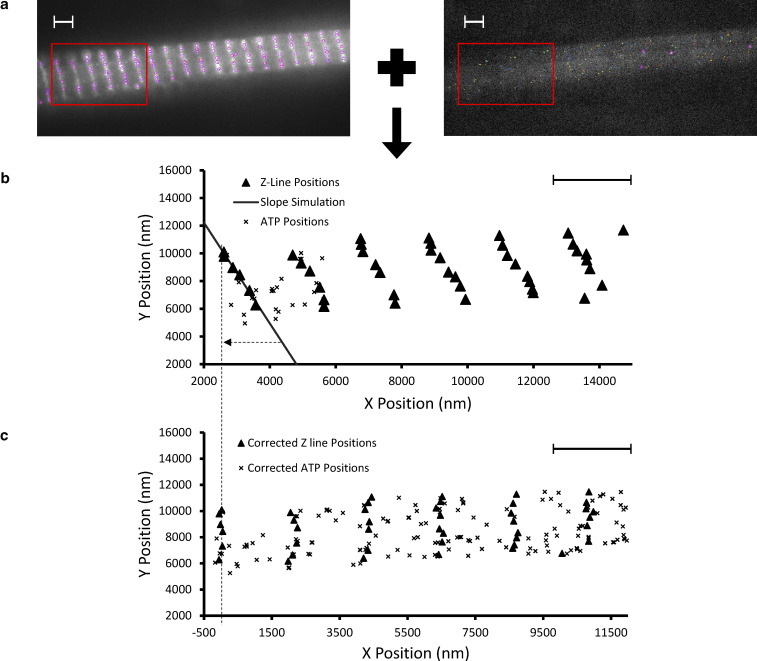
**Data analysis pipeline. (a)** The TrackMate analysis with Z-discs (left) and ATP localization events (right) as is seen during the analysis. Purple circles identify Z-disc tracked spots and multicoloured dots label Cy3-ATP tracks. **(b)** The combined data from both TrackMate analyses plotted with triangles representing the Z-disc positions and X marking Cy3-ATP localizations. A line of best fit was drawn through the tilted Z-disc and used to correct the tilt of that one sarcomere. This process was repeated for every sarcomere analyzed. **(c)** Corrected Z-disc and ATP localization data, with the dotted line showing the overlay of the axis with b; the aligned centre for the leftmost Z-disc becomes 0 on the x axis for c. All scale bars indicate 2 µm.

**Figure S4.**
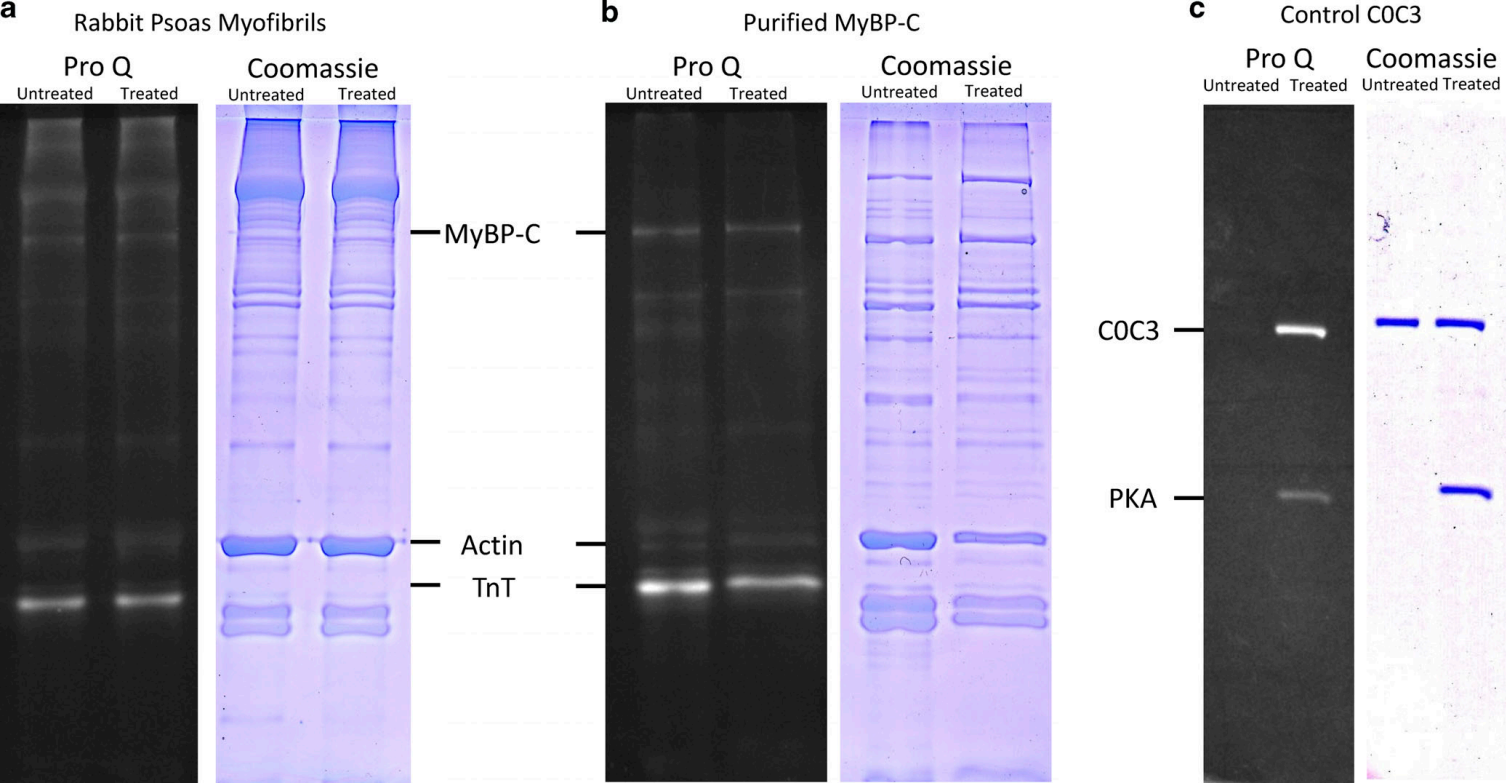
**The effects of PKA treatment on skeletal myofibrils. (a)** ProQ phosphostain- (left) and Coomassie-stained (right) images of myofibrils separated on SDS PAGE. **(b)** Purified MyBP-C samples similarly stained show no clear increase in ProQ staining after PKA treatment. **(c)** Control gel showing PKA activity on purified mouse C0C3, a ProQ clear band is seen post-PKA treatment, and the Coomassie gel confirms equal loading of protein sample. Untreated and PKA-treated samples are shown on left and right lanes, respectively, in all gels. Source data are available for this figure: SourceData FS4.

Fig. S1 describes the structure of the sarcomere with the thick filament zones indicated. Fig. S2 shows how time-lapse imaging reveals the contribution of photobleaching. Using time-lapse imaging, we can calculate the photobleaching rate constant, which then allows us to calculate the actual process rate constants. Fig. S3 shows the steps that are required for analyzing the data. Fig. S4 shows the effects of PKA treatment on skeletal myofibrils. This gel and blot show how PKA affects MyBP-C in skeletal myofibrils. Video 1 shows a combined view of the binding of individual Cy3-ATP molecules (cyan), overlaid with the α-actinin stained Z-discs (magenta). The positions of all bound ATP molecules were tracked. Supplemental text at the end of the PDF provides extended data analysis procedures, details of photobleaching controls performed, and data on the effects of PKA phosphorylation of myofibrils.

## Results

The three myosin states, active, DRX, and SRX, are distinguished by their kinetics. Using single-molecule imaging of fluorescent-ATP binding to rabbit psoas myofibril sarcomeres, we aimed to spatially resolve ATP turnovers of individual heads and hence ascertain where in the sarcomere these states exist and to what extent this distribution was affected by phosphorylation.

### Cy3-fluorescent ATP binds stoichiometrically to myosin

To confirm that Cy3-ATP binds to myosin (Toseland and Webb, 2011), we performed a stopped-flow experiment where rabbit myosin S1 was mixed with fluorescent ATP, and the change in tryptophan fluorescence at 320 nm was measured. Fig. 1 a shows the results of the titration of rabbit S1 against 500 nM (concentration after mixing) of Cy3-ATP. The amplitude data from stopped-flow transients provide a measure of the amount of ATP bound and were fit to a quadratic binding relationship to reveal an initial binding stoichiometry (from the E_T_ to S_T_ ratio) of 1.8 (±0.25) ATP:1 S1. The number of hydrolysis sites on S1 is just one, indicating that only half of the ATP was capable of binding to the heads. Therefore, we employed a pyruvate kinase/phosphoenolpyruvate-based regeneration system to convert any remaining ADP molecules back into ATP (Fig. 1 b, inset). Fig. 1 b shows the binding curve following Cy3-ATP regeneration, where the stoichiometry is now 0.92 (±0.14) ATP:1 S1. These data confirm previous observations that myosin hydrolyses Cy3-ATP normally (Toseland and Webb, 2011), therefore demonstrating it is not contaminated with ADP, validating its use in the single-molecule assay.

### Imaging individual ATP molecules binding to a myofibril reveals three lifetimes

In the absence of actin activation, ATP binds to a free myosin head and is rapidly hydrolyzed to ADP.P_i_ prior to P_i_ release, followed by ADP release. The duration of attachment at high ATP concentrations simplifies to the inverse sum of the lifetimes for each of these steps:

attachment duration=1hyd+1k−Pi+1k−ADP.

**Figure 2.**
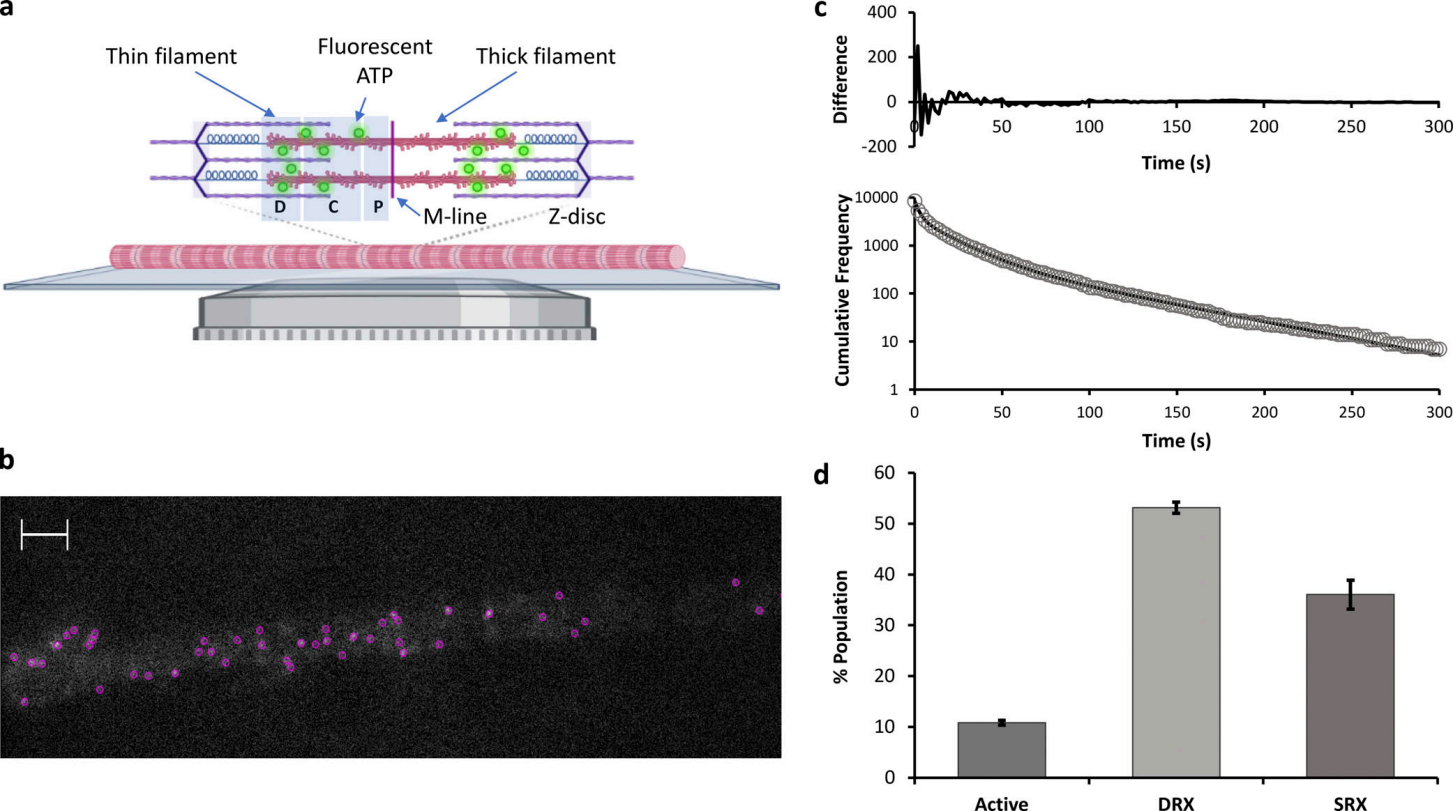
**Visualizing individual Cy3-ATP molecules binding and releasing from myofibrils. (a)** Using fluorescently tagged ATP (Cy3-ATP; untreated: n_events_ = 8,478, n_myofibrils_ = 9) microscopic imaging of individual myofibrils was performed on a custom-built imaging system where the illumination laser path is offset in the back focal plane to generate an oblique angle illumination field (OAF). Also shown is the structure of the sarcomere, where the D, C, and P zones are shaded in only one half sarcomere; this structure is also present on the other side of the M-line. **(b)** This system provides images of ATP binding and release events (Video 1) that are tracked using TrackMate (ImageJ). Individually bound ATP molecules are shown in magenta in the image. Scale bar indicates 2 µm. **(c)** All lifetimes are plotted as a cumulative residence time histogram and fitted to three exponentials (residuals shown in the inset). **(d)** The resulting amplitudes correspond to the different myosin states are normalized according to their rate constant (see Materials and methods).

The SRX states possess ATP turnover rate constants that are 10–100-fold slower than the DRX state (Nag et al., 2017). Therefore, this should appear directly as a longer duration of attachment. To assess the overall contribution from the SRX state, we imaged individual rabbit psoas myofibrils attached to a glass coverslip using obliquely inclined laser illumination at 561 nm (Fig. 2 a and Video 1) to reduce background from free Cy3-ATP in solution (Kad et al., 2010). To retain a relaxed state of the myofibril, we ensured the total [ATP] remained at 5 mM and pCa < 9; however, to enable visualization of individual fluorescent ATP molecules only 5 nM of the total ATP was Cy3-ATP. At this 1:1 × 10^6^ ratio of labeled to unlabeled ATP, we could observe individual bound Cy3-ATP localizations spatially and temporally separated from other bound Cy3-ATPs, crucial for super-localization microscopy. To obtain an overview of the SRX:DRX ratio, we captured images every 2 s but only illuminated for 200 ms of this period. This limited photobleaching enabled much longer visualization of the myofibril. We used TrackMate (ImageJ) to extract the locations and durations of individual bound Cy3-ATP (Fig. 2 b). The resulting lifetimes were plotted as cumulative residence time histograms (Fig. 2 c) and optimally fitted to three exponential decays. These decay constants were attributed to active, DRX, and SRX heads based on previous studies (Hooijman et al., 2011; Nag et al., 2017; Nelson et al., 2020). However, the observed ATPase rate constants from Fig. 2 c are convoluted with photobleaching, making estimates of the SRX rate constant likely an underestimate. To determine the effect of photobleaching, we used a method previously devised for imaging single molecules in cells (Gebhardt et al., 2013), and this approach adjusts the period between images while keeping the illumination time the same. Using this approach, it is possible to generate a known relationship between the photobleaching and attachment rate constants (Gebhardt et al., 2013):

$$k_{off}=k_{obs}-k_{bl}.\frac{\tau_{\exp}}{\tau_{tl}}\text{,}$$

where *k*_*off*_ is the actual rate constant for release, *k*_*obs*_ is the observed rate constant, *k*_*bl*_ is the photobleaching rate constant, *τ*_*exp*_ is the laser illumination time, and *τ*_*tl*_ is the period between images (time-lapse).

**Video 1.** **Cy3-ATP binding to a myofibril.** This video shows a combined view of the binding of individual Cy3-ATP molecules (cyan), overlaid with the α-actinin–stained Z-discs (magenta). The positions of all bound ATP molecules were tracked. Playback speed is 0.2 frames/s, and the Z-discs are ∼2 μm apart.

Time-lapse imaging was achieved using a custom Arduino-based camera/laser controller. The controller linked the timing of the camera to that of the laser pulse and enabled any configuration of illumination versus camera timing to be established. We determined the photobleaching rate constant for the slowest exponential since it is the most affected (Fig. S2), and therefore provided the most accurate measure. With this value, we could then correct for the impact of photobleaching on all of the measured rate constants. The relationship between the frame interval and the observed decay constant reveals the photobleaching rate constant of 0.13 s^−1^, which is used to correct the observed rate constants at the 2-s frame interval. The three corrected decay rate constants were 0.36 s^−1^, 0.04 s^−1^, and 0.004 s^−1^ for active, DRX, and SRX, respectively, consistent with previously reported values (Hooijman et al., 2011). This photobleaching correction was applied exclusively to make a comparison to the literature and was not used for the amplitude correction detailed below.

Finally, the amplitudes of each phase, which should reveal the relative populations, were corrected based on their frequency of occurrence. Faster rate constants would lead to greater turnover of ATP molecules and therefore more observed events. To account for this, we normalized the data with reference to the rate constant of the DRX population for each zone using the following equation:

$$AMP_{corr}=AMP_{fit}\frac{k_{DRX}}{k_{fit}}\text{,}$$

where *AMP*_*corr*_ is the corrected amplitude for the state, *AMP*_*fit*_ and *k*_*fit*_ are the fitted amplitudes and rate constants, respectively, for that state, and *k*_*DRX*_ is the fitted rate constant for the DRX population. Normalization was performed using DRX as the reference state, but any state could have been used with the same result. The percent of heads in a specific ATP hydrolysis state was then calculated using the corrected amplitudes. All amplitude normalization was performed using uncorrected rate constants (i.e., not photobleach corrected) for each state. After normalization, the relative contribution of each state to the total myofibrillar ATPase (Fig. 2 d) was determined as 10.8 (±0.5)% active, 53 (±1.1)% DRX, and 36 (±2.8)% SRX. It is important to note that although we assign the fastest population as active, this population does not necessarily refer to the active myosin state; it encompasses the binding of Cy3-ATP to any ATPase in the myofibril. However, we report this throughout as active for complete data transparency.

### Spatial deconvolution of attached lifetimes

**Figure 3.**
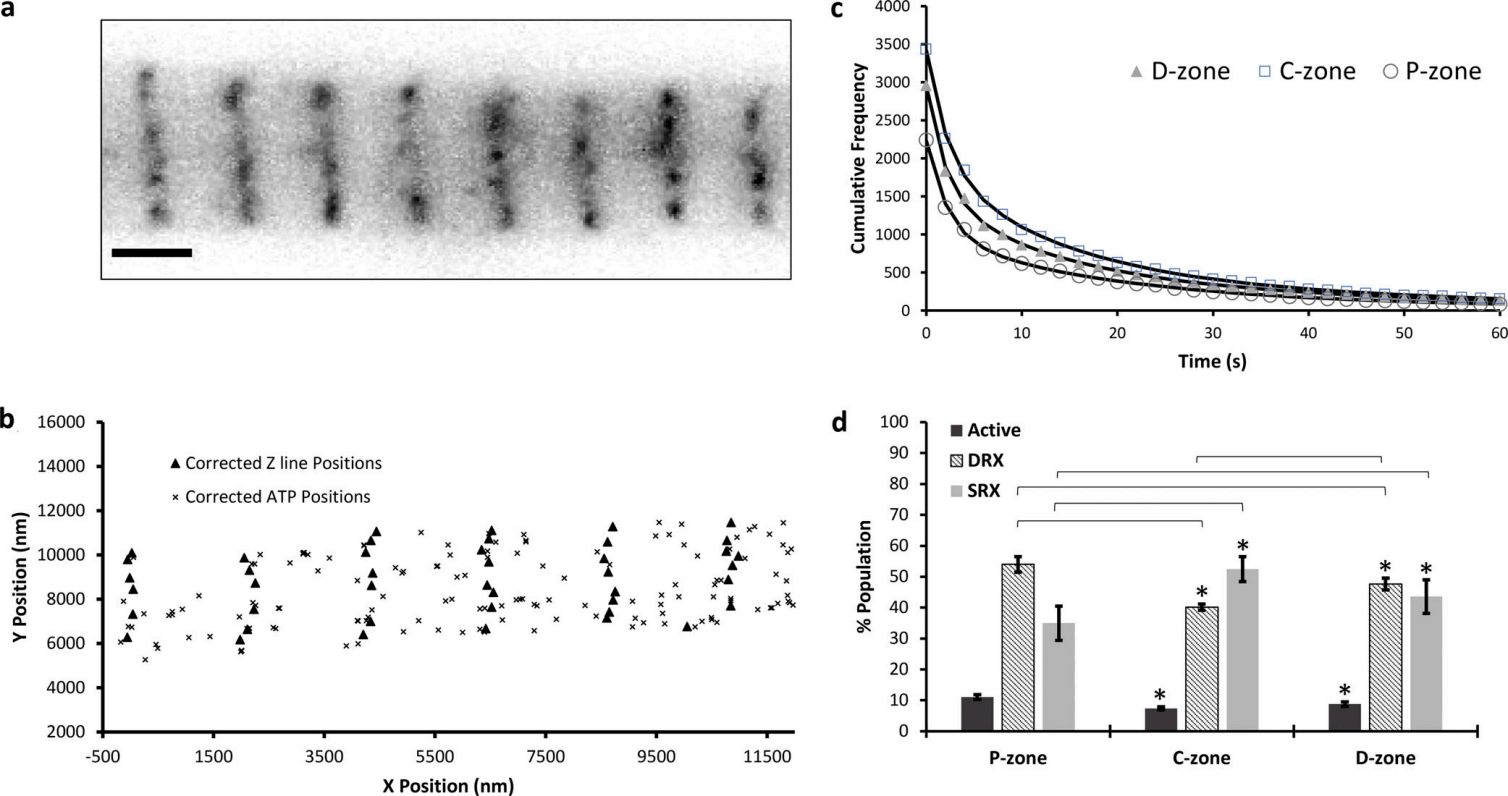
**Determining the location of the bound Cy3-ATP localizations within thick filament zones. (a)** Using fluorescently tagged anti–α-actinin antibodies the Z-discs are imaged. Scale bar indicates 2 µm. For greater accuracy, single-molecule localization is used to determine individual antibody locations, which are then used to delineate the sarcomere with 32 nm precision. **(b)** Individual Cy3-ATP localizations were superlocalized (precision was 29.2 nm in X and 26.7 nm in Y) using TrackMate and plotted relative to the locations of the Z-discs corrected to the horizontal axis (Fig. S3). Using known EM data, the boundaries of the P-, C-, and D-zones were used to bin bound Cy3-ATP localizations. All localizations outside of these zones were discarded. **(c)** The localization lifetimes were plotted as cumulative residence times. **(d)** Fitting these to triple exponential decays provides amplitude data to give the relative populations of each myosin state within each thick filament zone (see Table 1 for values). Significant differences (P < 0.05) between corresponding populations relative to the P- and C-zones are indicated with *. All C-zone populations: active (P = 0.0018), SRX (P = 0.0218), and DRX (P = 0.0001) are significantly different from the P-zone. For the D-zone: active (P = 0.04) and DRX (P = 0.0437) populations are significantly different from the P-zone. The only significant difference between D-zone and the C-zone populations is DRX (P = 0.0003).

Recent super-resolution studies have made great progress in identifying the locations of components in each region of the highly organized sarcomere (Tonino et al., 2019; Bennett et al., 2020), building on the magnificent work using electron microscopy (Craig and Offer, 1976). As stated above, TrackMate localization provided both temporal and spatial information for bound Cy3-ATP molecules. Therefore, we can assign lifetimes to subsarcomeric structures. Firstly, as a fiducial marker, we located the Z-discs using a fluorescently tagged antibody to α-actinin (Fig. 3 a). This was localized using TrackMate and aligned to the horizontal plane (see Materials and methods and Fig. S3) prior to extracting all ATP binding locations for each sarcomere (Fig. 3 b). By performing this task at the individual sarcomere level, we retained accuracy that would be lost in myofibrils that were not perfectly straight. Once aligned, we extracted the positions of the ATP localizations and binned them into the known P-, C-, and D-zones of the sarcomere (Tonino et al., 2019; Bennett et al., 2020). Any localizations outside these zones were discarded since they were likely due to other ATP binding proteins, for example, those located in the Z-disc; altogether these represented a small proportion (∼6%) of all detected ATP molecules. The positions of these zones was found to be similar between rabbit psoas (Bennett et al., 2020), mouse heart (Tonino et al., 2019), and rat soleus (Li et al., 2019; Nelson et al., 2020); therefore, for this study we used P-zone (0–159 nm), C-zone (160–500 nm), and D-zone (501–800 nm). The lifetimes of each ATP-bound event were plotted in a cumulative residence time histogram (Fig. 3 c), and the amplitudes of these fits used to calculate the populations of active, SRX, and DRX in these sarcomere zones (Fig. 3 d and Table 1).

**Table 1.** Percent populations of myosin active states by thick filament zone

Untreated (Fig. 3 d)			
**% (±SEM)**	**P-zone**	**C-zone**	**D-zone**
Active^a^	11.1 (±0.8)	7.4 (±0.5)	8.8 (±0.8)
DRX	54 (±2.5)	40.1 (±1)	47.7 (±1.9)
SRX	35 (±5.5)	52.5 (±4)	43.6 (±5.4)
**Mava (** Fig. 4 c **)**			
Active^a^	12.8 (±1.3)	6.7 (±0.4)	6 (±0.2)^b^
DRX	53.9 (±3.7)	33.4 (±1.1)^b^	30.6 (±0.6)^b^
SRX	33.3 (±10.8)	59.9 (±3.6)	63.4 (±2.7)^b^
**PKA (** Fig. 4 d **)**			
Active^a^	10.3 (±0.7)	5.7 (±0.3)^b^	8.1 (±0.4)
DRX	67.1 (±1.6)^b^	60 (±1.4)^b^	47 (±0.9)
SRX	22.6 (±6.6)	34.3 (±3.3)^b^	44.9 (±2.7)
**Change from untreated to mava (** Fig. 4 a **)**			
Active^a^	1.7 (±1.6)	−0.7 (±0.6)	−2.8 (±0.8)
DRX	−0.1 (±4.5)	−6.7 (±1.5)	−17.1 (±2)
SRX	−1.6 (±12.2)	7.4 (±5.4)	19.8 (±6.1)
**Change from untreated to PKA (** Fig. 4 b **)**			
Active^a^	−0.8 (±1.1)	−1.7 (±0.6)	−0.7 (±0.8)
DRX	13.1 (±3)	19.9 (±1.7)	−0.7 (±2.1)
SRX	−12.4 (±8.6)	−18.1 (±5.2)	1.4 (±6)
**SRX:DRX ratio (** Fig. 4 e **)**			
Untreated	0.6 (±0.1)	1.3 (±0.1)	0.9 (±0.1)
+Mava	0.6 (±0.2)	1.8 (±0.1)^b^	2.1 (±0.1)^b^
+PKA	0.3 (±0.1)	0.6 (±0.1)^b^	1.0 (±0.1)

a Active does not refer to active myosins necessarily. Instead, this simply refers to the fastest fitted phase which is likely dominated by nonspecific immobilizations of Cy3-ATP.

b Statistically significantly different from untreated (unpaired two-tail *t* test, P < 0.05).

### Perturbing the distribution of myosin’s states

**Figure 4.**
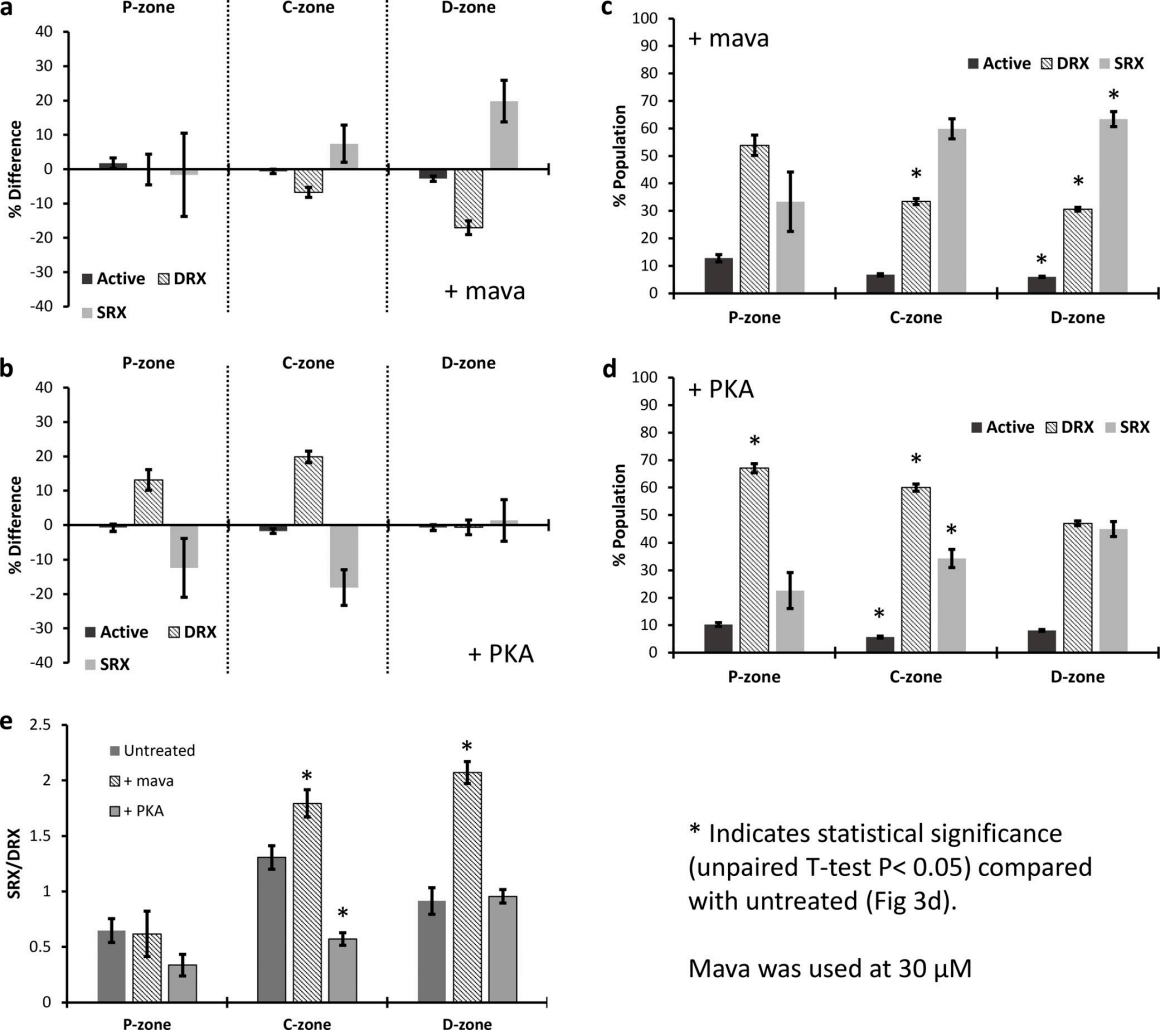
**Mava and PKA specifically alter the relative populations of SRX states in different thick filament zones. (a and c)** In the presence of mava (a), the populations of active, DRX, and SRX myosins are plotted relative to untreated thick filaments (n_events_ = 3,315, n_myofibrils_ = 11) and also as (c) percent populations within each zone. A significant difference in the DRX population of the C-zone (P = 0.0003) and in each population of the D-zone (active P = 0.0013, DRX P = 0.0001, SRX P = 0.0028) was observed. **(b and d)** With PKA (n_events_ = 2,705, n_myofibrils_ = 20), the changes relative to untreated are shown in b, whereas d shows the percent populations within each zone. See Table 1 for values. Significant changes were observed in all populations of the C-zone (active P = 0.0101, DRX P = 0.0001, SRX P = 0.0034), but only for the DRX in the P-zone (P = 0.0001). **(e)** To better represent the shift between DRX and SRX populations, the ratio of SRX:DRX is plotted revealing a clear significant effect of mava (P = 0.0093) and PKA (P = 0.0001) in the C-zone, whereas only mava (P = 0.0001) and not PKA affects the ratio of heads in the D-zone. Asterisks denote significant differences in values from untreated (Fig. 3 d), determined using an unpaired two-tailed *t* test (P < 0.05). The number of myofibrils was used as *n* values during statistical analysis.

The relative populations of the SRX and DRX states can be modulated by many factors, including temperature, force, and pharmacological interventions (Heling et al., 2020). Mava has been shown to increase the population of the SRX states by slowing P_i_ release (Green et al., 2016; Kawas et al., 2017). Using a high concentration (30 µM) of mava, we imaged the binding locations and lifetimes of ATP to provide the relative populations of states captured using a 2-s frame interval. Fig. 4 a shows the change in the relative proportions of SRX between P-, C-, and D-zones in the presence of mava. Zones C and D showed a significant decrease in the population of the DRX state and an increase in the SRX state. This is particularly pronounced in the D-zone, which is the second largest contributor of myosin crowns (∼6, 25, 18 in P-, C-, and D-zones, respectively; Bennett et al., 2020). The larger increase in D-zone SRX heads may reflect that few of the heads are in SRX relative to the C-zone (Fig. 3 d), leading to a statistically similar (P = 0.45) population of SRX heads (63% versus 60%) in both zones after mava treatment (Fig. 4 c).

In addition to studying mava, we also investigated the effects of cAMP-dependent kinase (PKA) phosphorylation on the relative populations of the myosin states. To phosphorylate myofibrils, we followed the protocol of Tong et al. (2008) and then studied the locations and durations of individual Cy3-ATP binding events. PKA phosphorylation affected the population of DRX myosin heads specifically in the P- and C-zones, with a significant increase in DRX appearing to draw from the SRX population (Fig. 4, b and d). Remarkably, no significant change in SRX or DRX was observed in the D-zone. To more clearly compare the data, we replotted the populations as the SRX:DRX ratio (Fig. 4 e). Using this view, neither treatment had a statistically significant effect on the P-zone whereas the addition of mava led to significant increases in the ratio for the C- and D-zones. The reduction in SRX:DRX ratio as a result of PKA treatment was only observed in the C-zone. These results are summarized in Table 1.

In addition to the changes in myosin states, we also observed a significant drop (unpaired *t* test) in the mean sarcomere length from 2,219 (±168 SD, P = 0.0319) nm for untreated to 1,920 (±377 SD) nm with PKA, compared to a negligible decrease in mean sarcomere length to 2,121 (±132, P = 0.2433) nm with mava.

## Discussion

Myosin in the thick filament of striated muscle that exists in three biochemical conformations: active, DRX, and SRX. During active contraction, the equilibrium between these states is shifted toward the active, high ATPase state. When relaxed, the ATPase activity of the sarcomere will decrease and the myosin heads primarily occupy the SRX and DRX states. How this equilibrium is regulated remains an open question, but phosphorylation of MyBP-C and/or light chains is implicated in this process. The high level of structural organization within the sarcomere restricts MyBP-C to the C-zone; therefore, by using single-molecule imaging it is possible to assign the location of individual ATPase events to any of the zones within the thick filament. By performing such an analysis, we see a high population of the SRX state in the C- and D-zones. Upon treatment with mava, which enhances the SRX state, a switch toward SRX from DRX is observed in the C- and D-zones. However, using the kinase PKA, we observed a switch toward DRX from SRX, primarily located in the C-zone. The precise mechanism of action is not known; however, in fast skeletal muscle there is a substantial population of the phosphorylatable slow MyBP-C in the C-zone; therefore, it is likely this is the target for PKA phosphorylation; however, we cannot rule out that other proteins, e.g., titin, may contribute to this change. Altogether, the results presented here offer a direct view of how the thick filament behaves in response to effector molecules and provide direct insights on the role of MyBP-C phosphorylation in skeletal muscle.

### Mava cannot completely repress myosin activity

Mava binds to myosin and reduces its ATPase, which is thought to favor the interacting head motif (IHM) potentially leading to more SRX (Anderson et al., 2018; Gollapudi et al., 2021), although the link between IHM and SRX is not certain (Chu et al., 2021). In this study, we used 30 µM mava (approximately sixfold greater than the reported K_d_; Kawas et al., 2017; Scellini et al., 2021) to ensure strong repression of the myosin ATPase regardless of position on the thick filament. Mava decreased the DRX population from 40.1% (30 heads) to 33.4% (25 heads) in the C-zone, and in the D-zone a much greater drop in DRX was seen, from 47.7% (26 heads) to 30.6% (17 heads). This resulted in a concomitant increase in SRX heads (Fig. 4 e). Nelson et al. (2020), using the same methodology as described here but in rat tissue, reported no effect of mava on the C-zone, instead all reductions in DRX were drawn from the P- and D-zones. Our observations for rabbit psoas indicate the final amounts of SRX are similar in the C- and D-zones, but much like the Nelson et al. (2020) study, there was a limit to the effects of mava. At the very high concentration of mava used in our study, it was not possible to completely repress the DRX heads (Fig. 4 b); instead, ∼36% remain. This suggests that mava is not potent in fast skeletal muscle, further validating its use for treating cardiac tissue specifically, even at concentrations significantly higher than the K_d_ for cardiac myosin (Scellini et al., 2021). Future studies using cardiac muscle would reveal if the drug is capable of a complete repression of DRX in that tissue.

It is important to this study and those subsequently using this technology to acknowledge that further improvements to the correlation with MyBP-C in the C-zone would be useful. The present study uses high-resolution data from EM and super-resolution imaging (Li et al., 2019; Tonino et al., 2019; Bennett et al., 2020); however, the number of MyBP-C repeats defining the C-zone has been suggested to be fewer (Luther et al., 2008). Therefore, future studies would benefit from the determination of MyBP-C position within the same assay; super-resolving the positions of individual MyBP-C using affimers (Carrington et al., 2019) offers such an approach. This experimentally challenging approach would enable precise correlation on a molecule-by-molecule basis of the effects of MyBP-C on vicinal myosins’ ATPase.

### PKA activates repressed myosins in the C-zone of the thick filament

The most relevant targets of PKA phosphorylation in cardiac myofibrils are troponin I and MyBP-C (Colson et al., 2012a). In skeletal muscle, AMPK is the key troponin I kinase, not PKA (Sancho Solis et al., 2011; Gross and Lehman, 2016). Also, the system studied here is relaxed (pCa < 9), therefore, TnI phosphorylation is not relevant. In addition, myosin’s light chains are phosphorylated by a specialized kinase, not PKA (Colson et al., 2010). More relevant is MyBP-C phosphorylation, which in the cardiac system reduces its affinity for myosin and actin, resulting in a shift toward the DRX state from SRX (Colson et al., 2008; Zoghbi et al., 2008). The data collected in this study are generated from rabbit psoas myofibrils, which contain primarily fast MyBP-C (fMyBP-C; Bennett et al., 1986), for which evidence for PKA phosphorylation does exist but perhaps may only occur in certain conditions (Lim and Walsh, 1986; Ackermann and Kontrogianni-Konstantopoulos, 2011; Colson et al., 2012b). Nonetheless, fast skeletal muscle has been shown to be comprised of a mixture of slow (sMyBP-C) and fMyBP-C isoforms (Reinach et al., 1983; Weber et al., 1993); sMyBP-C can be PKA phosphorylated (Ackermann and Kontrogianni-Konstantopoulos, 2011), providing a likely candidate for the effects seen in this study. A recent investigation of rat EDL muscle (fast fiber) revealed a mixture of 42% fMyBP-C (MYBPC2), and the remainder of the MyBP-C was comprised of the slow isoform and its splice variants (Li et al., 2019). Exactly what fMyBP-C does in skeletal muscle has not been fully determined; however, it has been implicated in controlling the number of accessible heads available for generating force either directly or through interfilament force sensing (Irving, 2017). A knockout (Song et al., 2021) or knockdown (Li et al., 2016) of fMyBP-C leads to an impairment of the force-generating capacity of these muscles. These studies also revealed that fMyBP-C promotes ordering of myosin heads onto the thick filament backbone, although this is at odds with the reduced observed force when knocked out (Song et al., 2021). In vitro and fiber studies have provided evidence that fMyBP-C is capable of slowing shortening velocity (Hofmann et al., 1991; Lin et al., 2018), possibly through drag interactions with the thin filament (Walcott et al., 2015). An absence of available monoclonal rabbit psoas MyBP-C antibodies coupled with uncertainties over the roles of sMyBP-C splice variants prevent the assignment of the SRX levels in our study to specific MyBP-C isoforms. However, the clear effects of PKA phosphorylation are likely due to sMyBP-C relieving inhibition of myosin heads (Robinett et al., 2019). Without PKA, 53% of myosins (39 myosins) in the C-zone are in SRX, and this is similar to the level of sMyBP-C in fast skeletal fibers (Li et al., 2019). Upon PKA treatment, the amount of SRX decreased by 19% to 26 myosins. Gel-based analysis of the phosphorylation profile for purified MyBP-C (Jacques et al., 2008) revealed an interesting picture (Fig. S4); we found that MyBP-C untreated with PKA showed evidence of phosphorylation, and from our measurements the amounts were very similar to that treated with PKA. This raises the possibility of a presently unknown phosphorylation event occurring in another protein such as titin. Since the effects of PKA phosphorylation on the myofibril are clear and located on the C-zone, the most likely target is MyBP-C (Fig. 4, d and e). If true, this implies a small increase in the phosphorylation (e.g., at another site) can cause a substantial shift in the SRX/DRX ratio. Further complicating interpretation is the complex nature of sMyBP-C splicing, which leads to a distribution of populations with different molecular weights, making identification of phosphorylated MyBP-C in the gel more challenging. Regardless, the effects of PKA on the C-zone are clear, and future studies (see above) correlating precise MyBP-C positions and isoforms with myosin activity are warranted.

### Conclusions

Mava has been shown to be highly effective at reducing myosin activity in cardiac systems. Here, we show that in rabbit psoas muscle mava reduces myosin activity by increasing SRX myosins, but the level of activity repression is limited and not complete. This reduced effect of mava has been previously reconciled as being due to a greater K_d_; however, we show here by working at concentrations of mava approximately sixfold above the K_d_ that the effects are still only marginal. Therefore, this reinforces mava’s specificity for cardiac muscle and indicates that off-target effects on other muscle tissues are likely to be minimal. We also show that PKA phosphorylation results in a reduction in SRX across both P- and C-zones while having a negligible effect on the D-zone. Given that only one PKA target in the C-zone is known for skeletal muscle, MyBP-C, this suggests that MyBP-C in skeletal muscle acts as it does in cardiac muscle to repress myosin activity. These new insights using direct visualization of myosin activation states offer a powerful approach to understand the molecular mechanisms of muscle activation and relaxation and also the precise mechanism of action for current and future pharmacological agents.

## Supplementary Material

SourceData FS4 is the source file of Fig. S4.
