# Supplementary material for: Single-molecule imaging reveals how mavacamten and PKA modulate ATP turnover in skeletal muscle myofibrils
Source: J Gen Physiol. 2022 Nov 17;155(1):e202213087. doi: 10.1085/jgp.202213087 (PMC9674027; doi:10.1085/jgp.202213087)

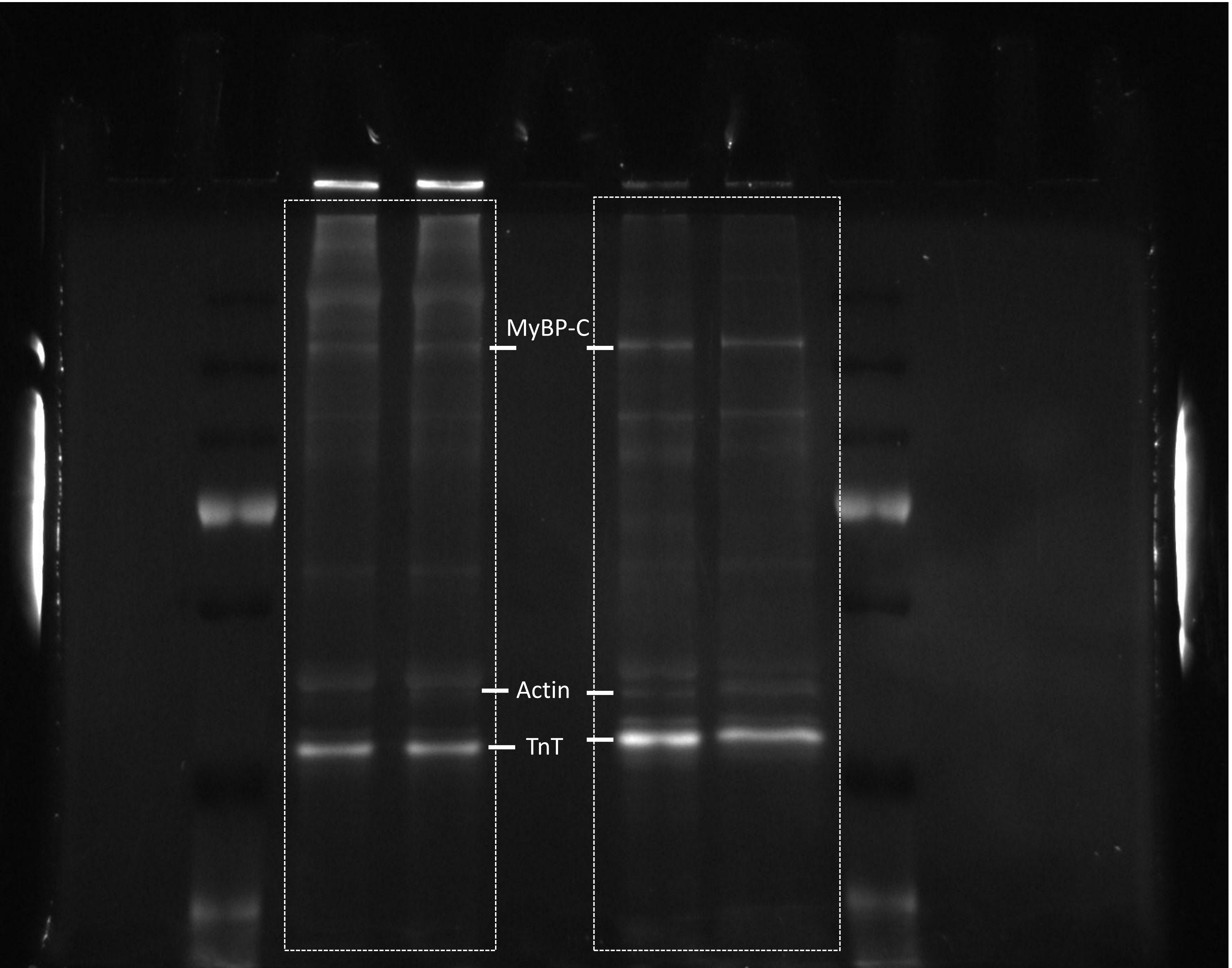


Figure S4A and Figure S4B ProQ gel source

Figure S4A and Figure S4B Coomassie gel source

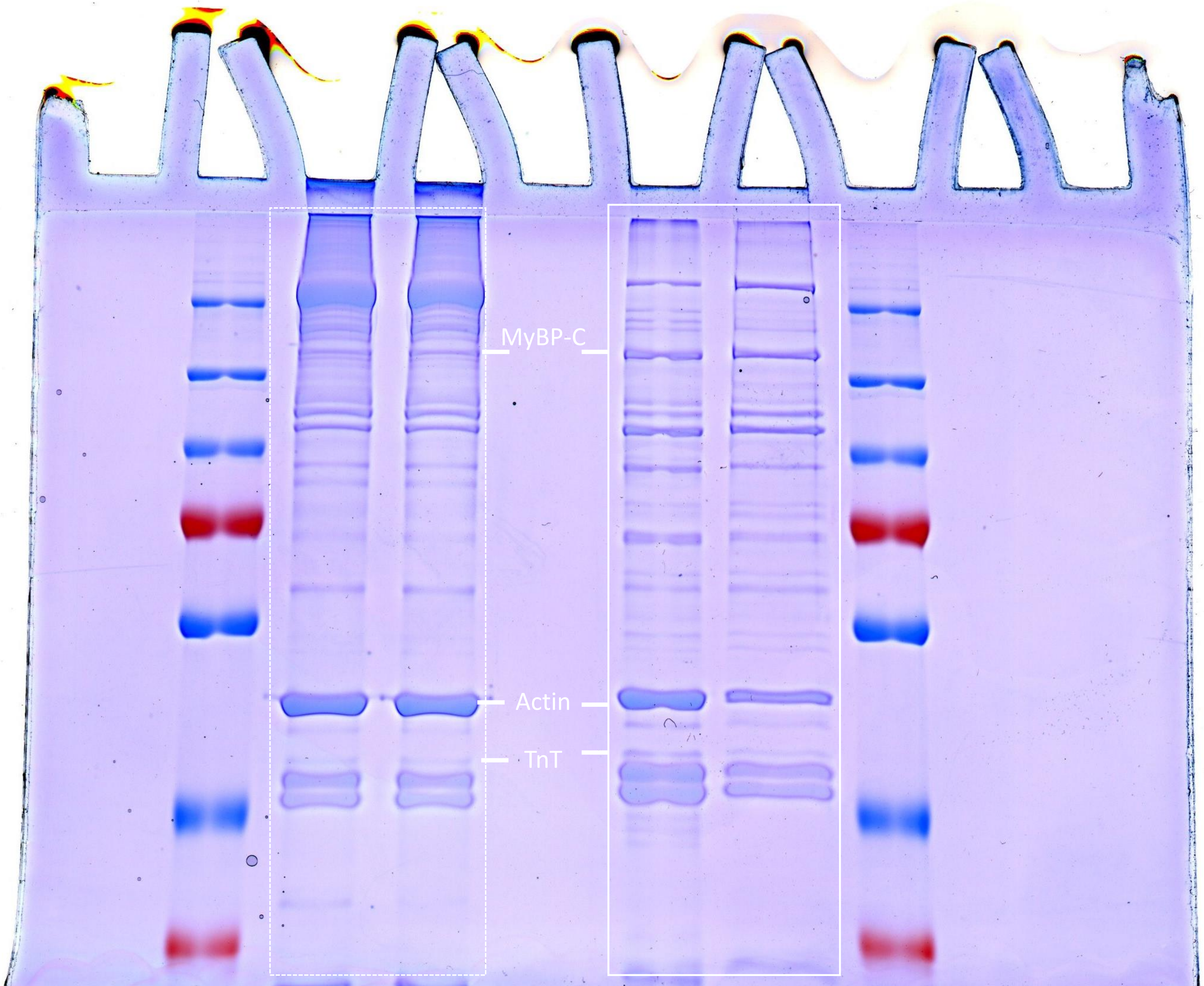

Figure S4C ProQ source gel

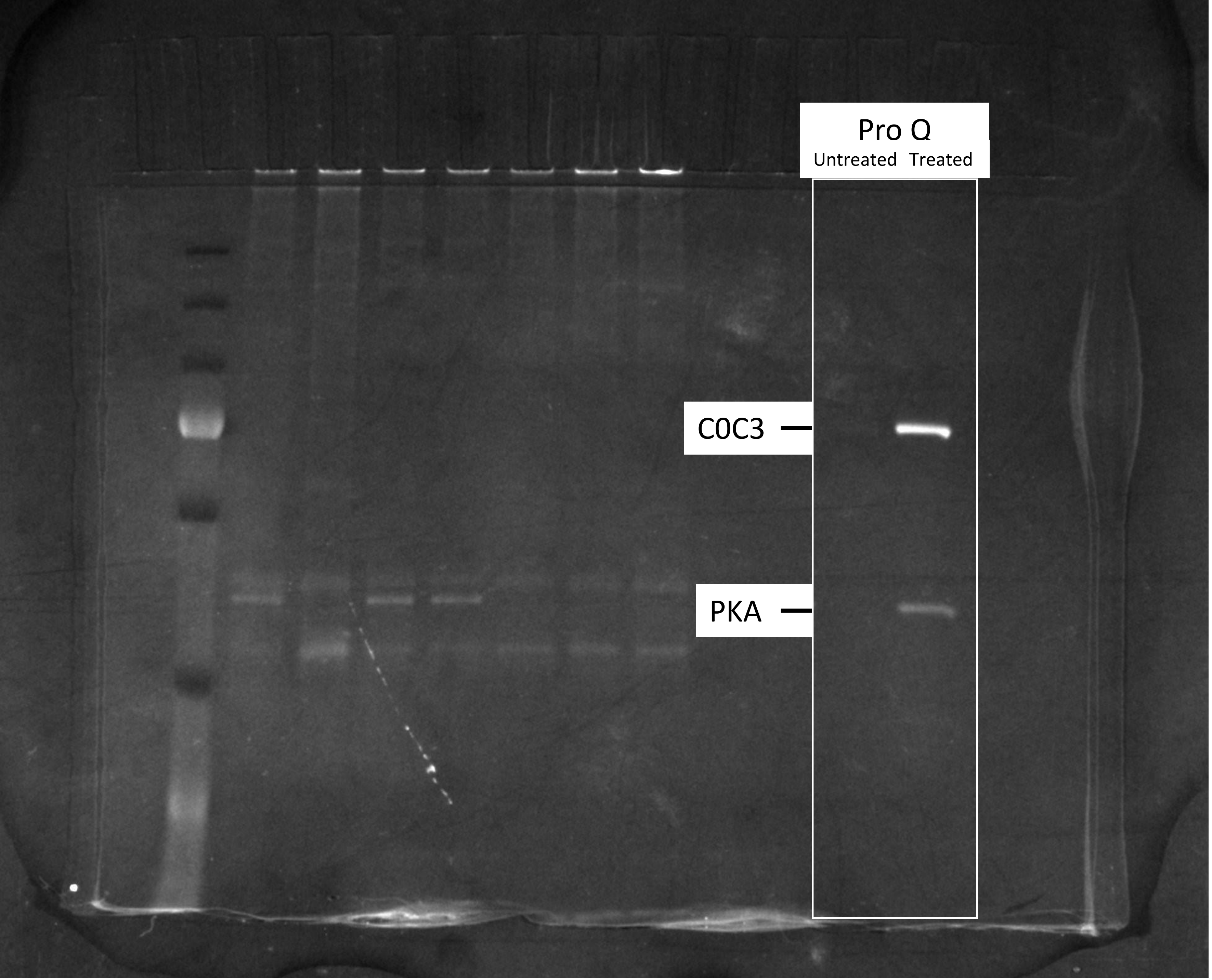

Figure S4C Coomassie source gel

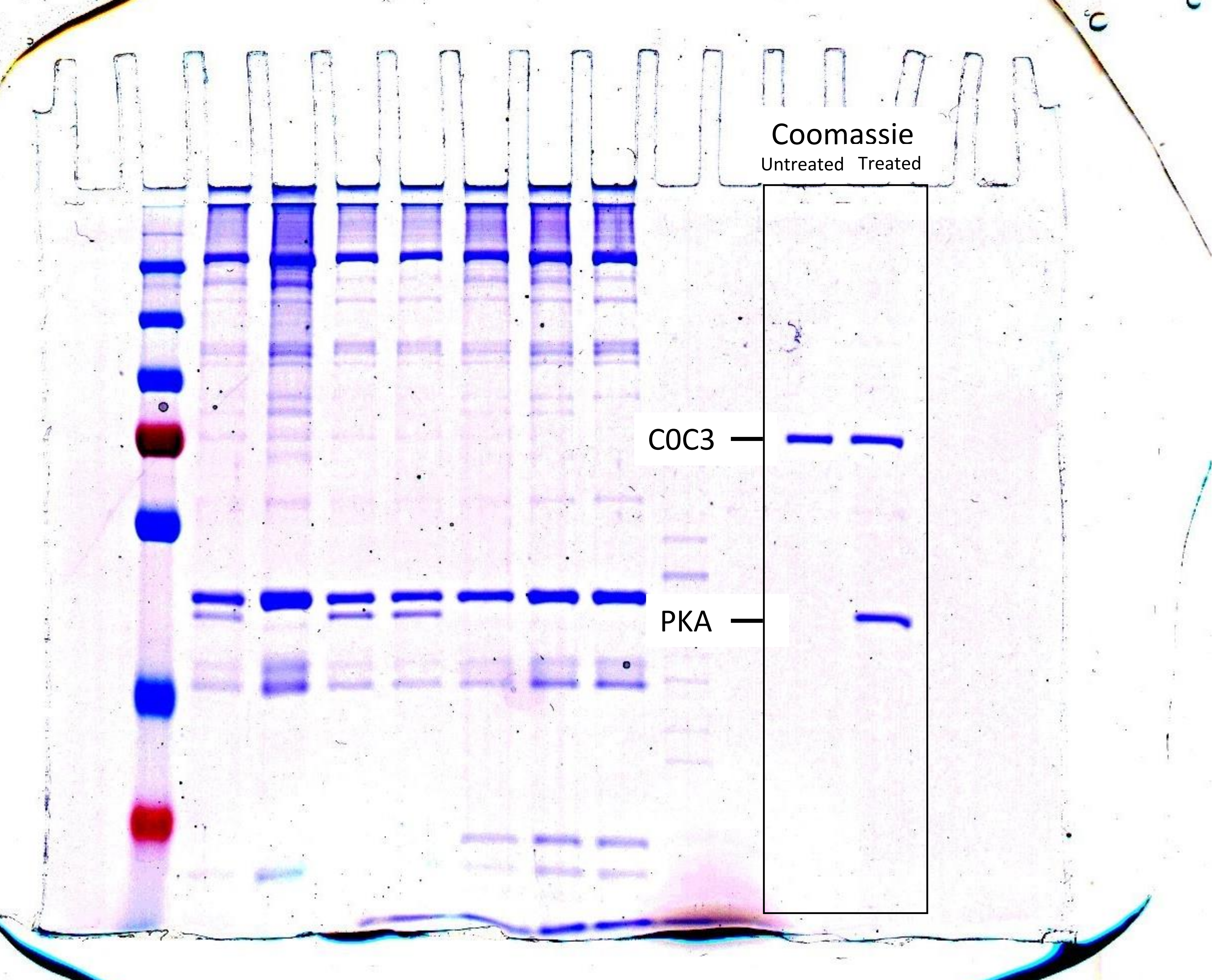

Supplement: SourceData FS4 — is the source file of Fig. S4. [file JGP_202213087_SourceDataFS4.pdf]
